# MicroED with the Falcon III direct electron detector

**DOI:** 10.1107/S2052252519010583

**Published:** 2019-08-17

**Authors:** Johan Hattne, Michael W. Martynowycz, Pawel A. Penczek, Tamir Gonen

**Affiliations:** a Howard Hughes Medical Institute, Department of Biological Chemistry, David Geffen School of Medicine, University of California, Los Angeles, CA 90095, USA; b Department of Biochemistry and Molecular Biology, The University of Texas McGovern Medical School, Houston, TX 77030, USA; c Howard Hughes Medical Institute, Department of Physiology, David Geffen School of Medicine, University of California, Los Angeles, CA 90095, USA

**Keywords:** microcrystal electron diffraction, MicroED, Falcon III, direct electron detectors

## Abstract

Microcrystal electron diffraction (MicroED) combines crystallography and electron cryo-microscopy (cryo-EM) into a method that is applicable to high-resolution structure determination. In MicroED, nanosized crystals, which are often intractable using other techniques, are probed by high-energy electrons in a transmission electron microscope. Diffraction data are recorded by a camera in movie mode: the nanocrystal is continuously rotated in the beam, thus creating a sequence of frames that constitute a movie with respect to the rotation angle. Until now, diffraction-optimized cameras have mostly been used for MicroED. Here, the use of a direct electron detector that was designed for imaging is reported. It is demonstrated that data can be collected more rapidly using the Falcon III for MicroED and with markedly lower exposure than has previously been reported. The Falcon III was operated at 40 frames per second and complete data sets reaching atomic resolution were recorded in minutes. The resulting density maps to 2.1 Å resolution of the serine protease proteinase K showed no visible signs of radiation damage. It is thus demonstrated that dedicated diffraction-optimized detectors are not required for MicroED, as shown by the fact that the very same cameras that are used for imaging applications in electron microscopy, such as single-particle cryo-EM, can also be used effectively for diffraction measurements.

## Abstract 2

MicroED data can be collected with the fastest and most sensitive cameras that are typically reserved for single-particle electron microscopy.

## 1. Introduction   

Microcrystal electron diffraction (MicroED) is a method in electron cryo-microscopy (cryo-EM) that exploits the strong interaction of electrons with matter to determine high-resolution structures from crystallized samples (Shi *et al.*, 2013 ▸). Owing to the favorable ratio of elastic to inelastic interactions (Henderson, 1995 ▸), MicroED can be used to collect useful data from crystals that are much smaller than are required for X-ray crystallography, for example. This is a significant advantage, since obtaining crystals that are both large and sufficiently well ordered to yield high-resolution diffraction data often constitutes a bottleneck in crystallo­graphy. Because crystals are screened and imaged using the same optical elements that are ultimately used to collect diffraction data, the large magnification of a transmission electron microscope (TEM) can be leveraged to select crystals with side lengths of as small as 50 nm (Rodriguez *et al.*, 2015 ▸). In contrast to single-particle cryo-EM, crystal constraints provide near-perfect alignment of the molecules and therefore the measured signal is strong enough to yield high-resolution structural information even from small peptides or chemical compounds (Gallagher-Jones *et al.*, 2018 ▸; Jones *et al.*, 2018 ▸; Ting *et al.*, 2019 ▸). MicroED thus provides a means to determine structures that are not attainable by other methods.

In MicroED, crystals are continuously rotated in the electron beam of a TEM and a fast camera is used to record a shutterless movie of the resulting diffraction patterns (Nannenga *et al.*, 2014 ▸). A camera for electron diffraction data collection must have a high dynamic range such that both low and high pixel values can be accurately recorded on the same frame: while the low-resolution spots may be strong enough to approach the upper limit of what a camera can measure, the high-resolution spots may barely be discernible over the background. Furthermore, under continuous rotation of the sample, the dead time during detector readout must be minimal, otherwise systematic gaps will be introduced in the sampling of reciprocal space.

While the majority of current TEMs in the cryo-EM field are equipped with sensitive direct electron detectors designed for imaging, these cameras have not been used for MicroED because of concerns over damage to the sensor by the intense incident beam as well as strong diffraction reflections. Instead, MicroED data have been collected using cameras that are not typically used for routine structure determination in other cryo-EM modalities, such as single-particle analysis and cryo-tomography. As a result, the number of MicroED practitioners has been limited because most facilities do not have the resources to provide a dedicated camera for MicroED. If MicroED data were to be collected using the very same direct electron detectors as are used for single-particle analysis, the number of laboratories with the ability to conduct MicroED measurements could increase substantially.

Here, we collected MicroED data from microcrystals of proteinase K using the Falcon III direct electron detector in integrating mode and compared the resulting structure with that obtained using the diffraction-optimized CMOS-based CetaD camera. Unlike the regular Ceta camera, which has previously been used for MicroED (Duyvesteyn *et al.*, 2018 ▸; Li *et al.*, 2018 ▸), the CetaD is fitted with a thicker scintillator to better capture the weak intensities of high-resolution Bragg spots (Martynowycz *et al.*, 2019 ▸). We demonstrate that reliable structure solution is possible from a typical direct electron-detecting camera, and that these cameras may even offer some advantages over those specifically designed for diffraction measurements. In order to facilitate this work, we developed the necessary software tools to convert data collected on the Falcon III and CetaD into images that can be processed in standard data-reduction suites such as *DIALS* (Winter *et al.*, 2018 ▸), *MOSFLM* (Leslie & Powell, 2007 ▸) and *XDS* (Kabsch, 2010 ▸). This software is freely available *via* our website (https://cryoem.ucla.edu/pages/MicroED).

## 2. Methods   

Proteinase K from *Engyodontium album* (Sigma–Aldrich, St Louis, Missouri, USA) was used without further purification to grow crystals in sitting drops (Hattne *et al.*, 2016 ▸). Protein powder dissolved in 50 m*M* Tris–HCl pH 8 was mixed with an equal amount of 1.25 *M* ammonium sulfate and dispensed into 24-well plates, where crystals appeared in less than 1 h. Sitting drops were diluted with well solution to a final volume of ∼25 µl, and crystals with typical side lengths of 2 µm and of <500 nm in thickness, all from the same batch, were placed on glow-discharged Quantifoil R2/2 Cu300 grids by pipetting 2 µl onto the carbon side. After blotting from the back for 5 s at 4°C and 100% environmental humidity, the grids were vitrified by plunging into liquid ethane and transferred into liquid nitrogen.

MicroED data were collected using an FEI Talos Arctica transmission electron microscope at an acceleration voltage of 200 kV using either a Falcon III or a CetaD as described below. For this work, Thermo Fisher disabled the diffraction protection on our Falcon III. The temperature was maintained at 77–100 K while samples were continuously rotated in the electron beam. A sequence of exposures, varying between 1 and 3 s in duration but constant for each of the six crystals (Table 1 ▸), were collected on the different cameras. In all cases the stage was rotated from high to zero tilt, but the rotation speeds were correspondingly higher for the three crystals imaged on the Falcon III (0.45° s^−1^) than for the crystals imaged on the CetaD (0.30° s^−1^). Care was taken to ensure that the standard beamstop was blocking the focused electron beam from striking the detector in diffraction mode, as exposure to the direct beam could damage the sensor. Data were integrated to the edge of the detector (2.1 Å for Falcon III, 2.3–2.8 Å for CetaD), and all crystals were measured with an estimated exposure rate of <0.01 e^−^ Å^−2^ s^−1^.

Images were converted from the native output format of the camera to SMV format using software developed in-house. The conversion programs extract the available metadata and automatically derive as much information as possible to allow downstream data-reduction packages to reconstruct the diffraction geometry; parameters not contained in the output (for example the calibrated sample-to-detector distance) must be specified by the user during image conversion. Since negative pixel values are retained in the native format they need not be explicitly modeled (Hattne *et al.*, 2016 ▸), but are addressed by adding a user-determined, per-data-set constant to all pixel values of each frame (here 8 ADU for data from the Falcon III and 128 ADU for CetaD frames). Pixel values below this pedestal (≤0.03% per Falcon III frame, ≤0.003% for the CetaD) were set to zero and discarded during integration. No further corrections were applied; in particular, procedures to correct for drifting dark current were disabled.

Data were indexed and integrated in *MOSFLM* (Leslie & Powell, 2007 ▸) using its graphical interface *iMosflm* (Battye *et al.*, 2011 ▸), taking the previously added pedestal into account. The gain *G* was estimated assuming a Poisson distribution of the background pixel values and was held fixed for both the Falcon III (*G* = 1.0) and the CetaD (*G* = 14). After scaling and merging in *AIMLESS* (Evans & Murshudov, 2013 ▸), the data were phased by molecular replacement in *MOLREP* (Vagin & Teplyakov, 2010 ▸) using PDB entry 5k7s (de la Cruz *et al.*, 2017 ▸) as a search model. Atomic models were refined in *REFMAC* (Murshudov *et al.*, 2011 ▸); automated solvent modeling and manual curation was performed in *Coot* (Emsley *et al.*, 2010 ▸). Custom analysis tools were written in Python using the *Computational Crystallography Toolbox* (*cctbx*; Grosse-Kunstleve *et al.*, 2002 ▸) and optimization routines implemented in *SciPy* (Oliphant, 2007 ▸).

## 3. Results and discussion   

We collected data from six crystals of proteinase K: three measured in integrating mode on the Falcon III and three on the CetaD (Table 1 ▸, Supplementary Videos 1 and 2). Both cameras were configured for 2 × 2 binning, yielding 2048 × 2048 pixel frames; for proteinase K diffracting to ∼2 Å resolution, binning resulted in a fourfold reduction of the data volume without causing detrimental spot overlap or loss of resolution. Indeed, unbinned data offer little advantage on the CetaD camera in MicroED, as the thicker scintillator is intended to trade spatial resolution for increased sensitivity. This modification is tailored towards diffraction measurements, where sensitivity is more important than spatial resolution.

Atomic resolution MicroED data were collected within minutes. Using both the Falcon III and the CetaD, several crystals were measured using an estimated exposure rate of 0.01 e^−^ Å^−2^ s^−1^ at an acceleration voltage of 200 kV. Because of the increased sensitivity of the Falcon III over the CetaD, the rotation speed for the Falcon III was 50% higher at 0.45° s^−1^ compared with 0.30° s^−1^ for the CetaD; the exposure time for the Falcon III was set to 1 s compared with the slower CetaD, which varied between 1.55 and 3.06 s per frame. Since the dose rate was identical, the Falcon III could record more information from a single crystal for the same total exposure: 129 frames were collected from each crystal on the Falcon III, whereas only up to 71 frames were collected on the CetaD.

The faster recording speed and lower exposures using the Falcon III contribute to make high-resolution data available for the duration of data collection. The resolution limit for the Falcon III data was 2.1 Å, compared with 2.3–2.8 Å for the CetaD (Table 1 ▸). In sharp contrast to both cameras, data previously collected from proteinase K on a TVIPS TemCam-F416 under otherwise similar conditions (Hattne *et al.*, 2018 ▸) used significantly longer exposures (4–5 s) with correspondingly slower rotation speeds (0.09° s^−1^) and higher total exposure.

With the Falcon III and the CetaD, data can be collected an order of magnitude faster compared with previous reports for proteinase K (Hattne *et al.*, 2016 ▸, 2018 ▸; de la Cruz *et al.*, 2017 ▸). The increased sensitivity of the Falcon III allows the per-frame exposure to be reduced during data collection because fewer electrons are required to obtain a sufficiently strong signal over the noise of the background. Combined with the higher readout rate, this implies that complete data sets can be collected both faster and using a lower total exposure than previously possible. While the precise relationship between exposure and absorbed dose depends on many factors, higher exposures always increase the absorbed dose. Since absorbed dose is directly related to radiation damage, the ability to obtain complete data sets with a lower exposure is expected to translate to final models of proteins that are less damaged.

**Figure 1:**
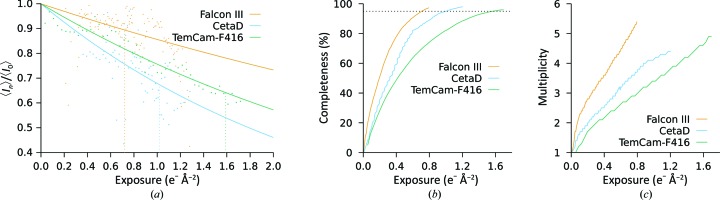
Mean intensity, completeness and multiplicity as a function of exposure. (*a*) For each camera, the integrated intensities on the subset of frames in the (−30°, +30°) tilt range were averaged; this reduces systematic effects on the intensities arising from longer paths through the sample at higher tilt. The reflections in the resolution range common to all data sets (20.0–2.70 Å) were then fitted to a function of the form *A*_cryst_ exp(−*B*_cam_ × *x*), where *A*_cryst_ was refined for each crystal and *B*_cam_ was refined for each camera. The dotted vertical lines indicate the exposure at which 95% completeness was obtained. (*b*) The exposure-dependency of the completeness was determined by merging only frames with an average exposure less than the given value. The dotted horizontal line marks 95% completeness. (*c*) In all cases, the multiplicity increases approximately linearly with dose, which implies that completeness is indicative of the amount of information recovered at the given dose.

One of the first noticeable effects of radiation damage is the exponential falloff of intensity with increasing exposure (Blake & Phillips, 1962 ▸; Baker & Rubinstein, 2010 ▸; Liebschner *et al.*, 2015 ▸; Hattne *et al.*, 2018 ▸), commonly characterized by the dose at which the average integrated intensity drops below some threshold. The rate of radiation-related intensity reduction is dependent on the sample, both its chemical composition and the size of the illuminated crystal (Nave & Hill, 2005 ▸), but should be unaffected by the detector. For proteinase K measured on the Falcon III and the CetaD, the intensity fell to 50% of its extrapolated value at zero dose when exposed to 2.5 and 1.6 e^−^ Å^−2^, respectively [Fig. 1 ▸(*a*)]. These values agree with *D*_50_ = 2.2 e^−^ Å^−2^ as previously found for the same sample measured on a TVIPS TemCam-F416 (Hattne *et al.*, 2018 ▸) and indicate that microscope and camera parameters are well calibrated.

In Figs. 1(*b*) and 1(*c*) ▸ we show comparisons of the number of reflections, given by completeness and multiplicity, integrated on diffraction patterns from different crystals and plotted as a function of exposure. While radiation damage ultimately limits the amount of useful information, the attainable completeness is generally restricted by the orientation of the crystal in the beam and is further constrained by the permissible rotation range, neither of which are under user control. The total dose cannot be arbitrarily reduced, as this will negatively impact the confidence of the measurement. For three crystals, a total exposure of 1.0 e^−^ Å^−2^ was needed to reach 95% completeness on the CetaD, whereas only 0.7 e^−^ Å^−2^ was needed for the Falcon III. In both cases, the multiplicity increases approximately linearly [Fig. 1(*c*) ▸], indicating that the completeness reflects the amount of information recovered at the given exposure. These exposures are both significantly lower than that of 1.6 e^−^ Å^−2^ previously required to reach the same completeness on the TVIPS TemCam-F416 (Hattne *et al.*, 2018 ▸).

**Figure 2:**
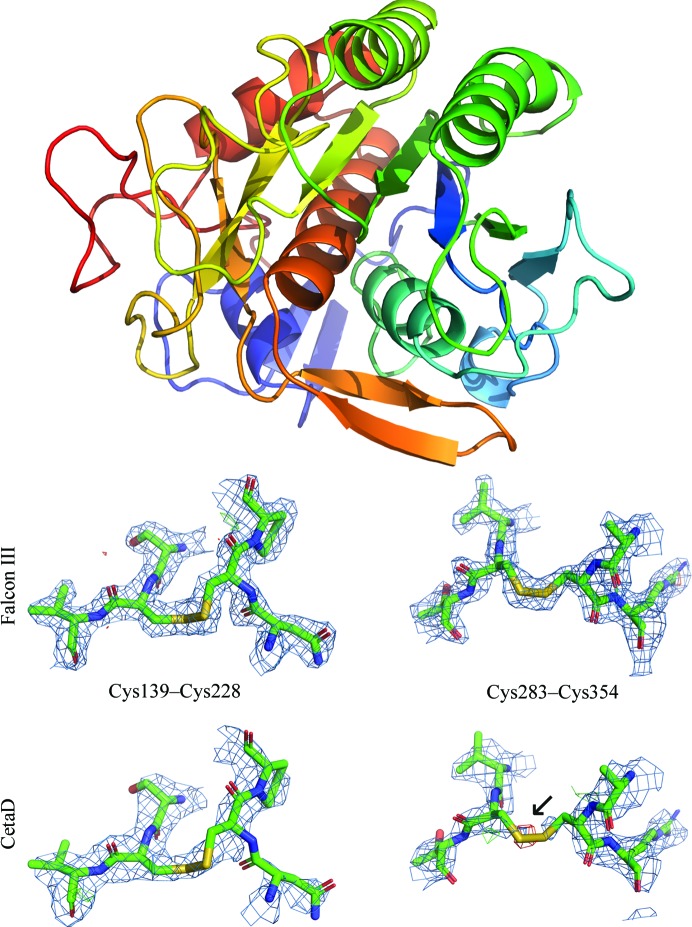
The structure of proteinase K determined from Falcon III data and the density around its two disulfide bonds for the considered cameras. The density around the two disulfide bonds indicates increasing radiation damage as an effect of increasing dose. The positive difference density around C^β^ of Cys283 in the CetaD data (black arrow) indicates a partially dislocated S atom. The 2*mF*_o_ − *DF*_c_ densities (blue meshes) are contoured at 1.5σ above the mean; *mF*_o_ − *DF*_c_ difference densities (green/red meshes) are contoured at ±3σ around the mean. All meshes were carved to 2 Å around the selected atoms in *PyMOL* (Schrödinger; https://www.pymol.org).

The manifestation of damage in real space similarly agrees with previous observations. In Fig. 2 ▸ we show the density around the two disulfide bonds in proteinase K, where damage to the Cys283–Cys354 bond is immediately apparent in the data collected on the CetaD (total exposure of 1.2 e^−^ Å^−2^). In comparison, the total exposure for the data collected on the Falcon III (0.8 e^−^ Å^−2^) is about two thirds of that for the CetaD data. At this level of detail, damage to the disulfide bonds cannot be observed in the data collected on the Falcon III.

**Figure 3:**
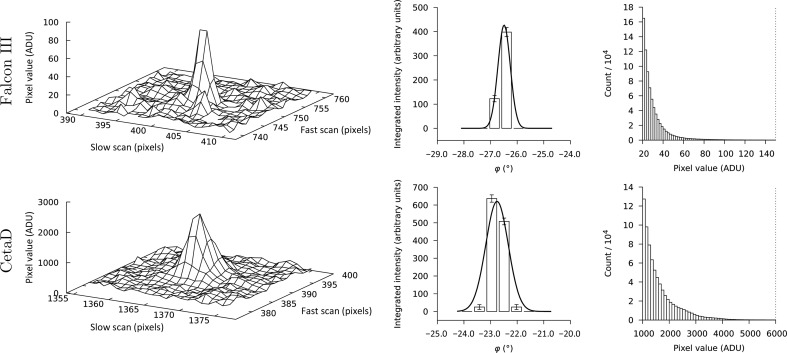
The (10, 16, 12) reflection at ∼3.3 Å resolution on consecutive frames and the distribution of integrated pixel values. Pixel values recorded on the Falcon III (top row; rotation speed dφ/d*t* = 0.45° s^−1^, Δφ = 0.45° per frame) and the CetaD (bottom row; rotation speed dφ/d*t* = 0.30° s^−1^, Δφ = 0.46° per frame) cameras. The central panel in each row shows the profile-fitted intensities as integrated by *MOSFLM*, where the error bars span one standard deviation of the integrated intensity and a Gaussian function, based on the progression of the reflection through the diffractive condition, has been fitted to aid the eye. For both the Falcon III and the CetaD, the pixel values in a 11 × 11 pixel box centered on the predicted spot locations are within the linear range of the detector, limited by the vertical dotted line (right panel). The physical pixel sizes on both cameras are identical (14 µm, square). Note that the peak counts on the CetaD are more than an order of magnitude higher than on the Falcon III, as the Falcon III in integrating mode reports the average pixel values of the individual frames at 40 Hz.

The diffraction spots measured on the Falcon III are generally sharper than those observed on the CetaD (Fig. 3 ▸, left panels). This is partially owing to differences between the measured crystals, but is also an effect of the more compact point-spread function of the Falcon III. In neither case do we observe systematically saturated reflections, not even at low resolution where reflections tend to be much stronger than at high resolution (Fig. 3 ▸, right panels). The linear range for pixels on single frames from both cameras extends to ∼6000 ADU per pixel and frame; pixel values on images from the Falcon III reflect the average of 40 frames, hence pixel values of <150 ADU are assumed to lie in the linear range.

## 4. Conclusion   

We have demonstrated that the typical direct electron detectors used for other cryo-EM modalities such as single-particle cryo-EM can also be used for MicroED, alleviating the need for additional dedicated cameras. Compared with cameras used previously, the Falcon III and CetaD offer the possibility of collecting complete data at lower exposures in a shorter amount of time. This has immediate implications for efforts to automate MicroED data collection, where the efficient use of shared resources may be a major concern, but also leads to structural models with reduced radiation damage. For example, combining MicroED data collection in *SerialEM* (de la Cruz *et al.*, 2019 ▸) with a Falcon III direct electron detector can result in the autonomous collection of more than 300 complete data sets overnight; this level of productivity is commensurate with X-ray crystallography at synchrotrons. As MicroED is gaining momentum in the cryo-EM field, which is already undergoing rapid changes, developing MicroED data-collection protocols and software analysis tools to optimally use new hardware will be a priority for the immediate future.

Other electron detectors have been used in MicroED applications previously. For example, hybrid pixel detectors such as the EIGER (Tinti *et al.*, 2018 ▸), Medipix (Nederlof *et al.*, 2013 ▸) or Timepix (van Genderen *et al.*, 2016 ▸) provide electron-counting capabilities and offer alternatives to the cameras discussed here in terms of dynamic range and frame rate. However, these cameras have shortcomings that we consider to be detrimental to macromolecular MicroED applications. Owing to their current small chip size and large physical pixel size, recording high-resolution MicroED data from macromolecules with large unit cells presents a challenge on these devices. On a 512 × 512 pixel detector, for instance, the spots on a diffraction pattern of the (*h*, *k*, 0) zone of proteinase K to 2.1 Å resolution would be separated by approximately eight pixels when the beam center is near the center of the detector. Assuming a spot diameter of ten pixels, this will result in frequent spot overlaps, which degrade integration accuracy and can lead to loss in resolution as longer camera lengths would have to be used to compensate for the lack of detector real estate. Furthermore, their large point spread currently makes these detectors unsuitable for high-resolution imaging, making them truly dedicated cameras for diffraction studies.

The higher sensitivity and readout rate of the Falcon III camera allow complete data sets to be collected faster, with higher precision and with lower total exposure compared with CMOS-based detectors. The average time to record a single data set from proteinase K was less than half of the time previously required to collect similar low-dose data sets (Hattne *et al.*, 2018 ▸). In fact, the average exposure for the data merged from the Falcon III is also less than half of that previously reported for the TVIPS TemCam-F416.

The Falcon III camera implements an electron-counting mode in addition to the integrating mode used to collect the data here. Electron counting has the potential for measuring data at near-optimal detective quantum efficiency (McMullan *et al.*, 2016 ▸), but requires the data collection to be carefully calibrated to maintain a maximum of one count per pixel per frame. The proper setup would require deriving a balance between specimen rotation speed, frame-readout rate and the total dose. Indeed, further work is necessary to make electron counting using the Falcon III a feasible mode of data collection for MicroED.

## 5. Software availability   

Software tools that convert the native output format, both MRC (Cheng *et al.*, 2015 ▸) and TIA series files, to Super Marty View (SMV) or TIFF are available at https://cryoem.ucla.edu/pages/MicroED and will be included in an upcoming release of the rebranded MicroED tools. The updated version also contains programs that support data collected with *SerialEM* (de la Cruz *et al.*, 2019 ▸).

## Supplementary Material

PDB reference: proteinase K, 6pu4

PDB reference: 6pu5

Supplementary Movie S1. Proteinase K recorded to 1.98 Å (edge) on a Falcon III camera. DOI: 10.1107/S2052252519010583/fq5007sup1.mp4

Supplementary Movie S2. Proteinase K recorded to 2.3 Å (edge) on a CetaD camera. DOI: 10.1107/S2052252519010583/fq5007sup2.mp4

## Figures and Tables

**Table 1:** Processing and refinement statistics for proteinase K recorded on the Falcon III and CetaD cameras *D* is the virtual sample-to-detector distance, which corresponds to the physical distance in an otherwise equivalent lensless system, and *t*_exp_ denotes the exposure time per frame during data collection. Note that not all collected frames were merged, and this is reflected in *E*_max_, the maximum exposure of any frame in the merged data set. Values in parentheses refer to the highest resolution shell for merging. All data were collected at an acceleration voltage of 200 kV.

	Falcon III (PDB entry 6pu4, EMDB entry EMDB-20475)			CetaD (PDB entry 6pu5, EMDB entry EMDB-20476)		
Data collection						
*D* (mm)	2380	2380	2380	3200	3200	2660
*t*_exp_ (s)	1.00	1.00	1.00	3.06	3.05	1.55
Rotation speed (° s^−1^)	0.45	0.45	0.45	0.30	0.30	0.30
Data processing						
Resolution (Å)	27.64–2.10 (2.16–2.10)			28.58–2.70 (2.83–2.70)		
*E*_max_ (e^−^ Å^−2^)	0.80			1.22		
*R*_merge_	0.479 (1.612)			0.440 (1.972)		
No. of observations	73941 (3646)			28788 (3163)		
No. of unique observations	13802 (1053)			6520 (825)		
〈*I*/σ(*I*)〉	3.1 (1.0)			3.6 (0.9)		
CC_1/2_	0.907 (0.269)			0.873 (0.169)		
Completeness (%)	97.3 (92.6)			98.0 (97.2)		
Multiplicity	5.4 (3.5)			4.4 (3.8)		
Refinement						
*R*_work_/*R*_free_ (%)	22.06/26.70			23.13/26.59		
R.m.s.d., bond lengths (Å)	0.0084			0.0078		
R.m.s.d., bond angles (°)	1.4723			1.4250		
